# The Emerging Role of Stress Granules in Hepatocellular Carcinoma

**DOI:** 10.3390/ijms22179428

**Published:** 2021-08-30

**Authors:** Dobrochna Dolicka, Michelangelo Foti, Cyril Sobolewski

**Affiliations:** Department of Cell Physiology and Metabolism, Faculty of Medicine, University of Geneva, CH-1211 Geneva, Switzerland; Dobrochna.Dolicka@unige.ch

**Keywords:** stress granules, liver diseases, hepatitis, Adenylate-Uridylate-rich element-binding proteins, oncogenes, tumor suppressors, post-transcriptional regulation

## Abstract

Stress granules (SGs) are small membrane-free cytosolic liquid-phase ordered entities in which mRNAs are protected and translationally silenced during cellular adaptation to harmful conditions (e.g., hypoxia, oxidative stress). This function is achieved by structural and functional SG components such as scaffold proteins and RNA-binding proteins controlling the fate of mRNAs. Increasing evidence indicates that the capacity of cells to assemble/disassemble functional SGs may significantly impact the onset and the development of metabolic and inflammatory diseases, as well as cancers. In the liver, the abnormal expression of SG components and formation of SG occur with chronic liver diseases, hepatocellular carcinoma (HCC), and selective hepatic resistance to anti-cancer drugs. Although, the role of SG in these diseases is still debated, the modulation of SG assembly/disassembly or targeting the expression/activity of specific SG components may represent appealing strategies to treat hepatic disorders and potentially cancer. In this review, we discuss our current knowledge about pathophysiological functions of SGs in HCC as well as available molecular tools and drugs capable of modulating SG formation and functions for therapeutic purposes.

## 1. Introduction

Hepatocellular carcinoma (HCC) is the seventh most common cancer and the second biggest cause of cancer mortality worldwide [1,2]. HCC can arise in the context of various chronic liver diseases, including chronic hepatitis B and C viral infections (HBV and HCV, respectively), alcoholic liver disease (ALD), and non-alcoholic fatty liver disease (NAFLD), a metabolic liver disorder tightly associated with obesity, diabetes, and sedentary lifestyle [1,2]. Most of these hepatic diseases start with the aberrant accumulation of fat in hepatocytes, a condition called steatosis, and are thus referred as fatty liver disease (FLD). With time, lipotoxicity, endoplasmic reticulum (ER) stress, and mitochondrial dysfunctions lead to hepatocyte death and inflammation (steatohepatitis) with the associated accumulation of fibrotic tissues in the liver [2,3]. If unresolved, inflammation and fibrosis can progress with time and regenerative nodules of poorly differentiated hepatocytes develop in the parenchyma leading to a loss of hepatic functions and portal hypertension [4]. This end stage of FLD is a life-threatening conditions per se, but also an important risk factor for HCC development [5]. Of note, HCC can also arise in non-cirrhotic conditions, directly from early stages of FLD (steatohepatitis/fibrosis) in the absence of cirrhosis [6]. Importantly, given the rapid worldwide increase of the prevalence of NAFLD with obesity and diabetes [7] and the high prevalence of ALD in developed countries, HCC incidence is expected to dramatically increase in the future [8], thus representing a major public health concern and an economic burden. HCC is one of the less curable cancers, due to the limited number of available therapeutic options and the high resistance of this cancer to conventional chemotherapy and radiotherapy. Surgical resection or liver transplantation remain the most efficient strategies but not all patients are eligible for surgery and these interventions are associated with life-threatening issues [9,10]. Understanding the molecular mechanisms of FLD and HCC is therefore required to develop new and efficient preventive and therapeutic approaches.

A myriad of molecular alterations is associated to the development of FLD and HCC, among which abnormal post-transcriptional regulation of gene expression is a key pathological mechanism driving hepatic metabolic diseases and carcinogenesis. Alterations of microRNAs (miRNAs) and RNA-binding proteins (RBPs) expression and/or activity promote mRNA decay or impair translation of transcripts controlling hepatic metabolism, inflammation, and carcinogenesis [11]. Similarly, to genetic mutations, alterations of these post-transcriptional regulators of gene expression may lead to an overexpression of oncogenes or the silencing of tumor suppressors, thereby favoring cancer development. Some of these regulatory mechanisms take place within small cytoplasmic ribonucleoprotein foci, such as processing bodies (P-bodies) or stress granules (SGs), where the fate of mRNAs is determined (i.e., translation, degradation, etc.) [12]. SGs, like P-bodies, are small cytosolic compartments devoid of membranes and containing translationally stalled mRNAs [13,14,15]. SGs form in stress conditions (e.g., nutrient deprivation, hypoxia), likely to protect mRNAs from degradation and spare energy to re-synthesize them when required again [14]. Consistent with this function, SGs contain several components of the translation initiation complex (e.g., 40S subunit). P-bodies share similar biophysical properties (liquid–liquid phase separation) and common protein components with SGs (e.g., tristetraprolin, BRF1) [16]. However, P-bodies differ from SG by the presence of several factors triggering mRNA decay, such as decapping enzymes (e.g., DCP1A, EDC4), deadenylases (i.e., Ccr4-Not, Lsm1-7), and exonucleases (e.g., Xrn1) [16]. P-bodies may be present in physiological conditions but increase in number and size during cellular stress [16]. Several stress stimuli promote the formation of both P-bodies and SGs (e.g., heat shock or oxidative stress), while others (e.g., viral infection) are specific for SGs [16].

The molecular mechanisms governing SG assembly are still incompletely understood, but the interaction of several RBPs with mRNAs appear to be determinant [13,14]. SG formation is usually reversible, and in case of prolonged stress, mRNAs located within SGs can be translocated and degraded within P-bodies [12]. The biogenesis and stability of SGs have been associated to the development of a wide range of diseases and cancers [14]. Consistent with a functional relevance of SGs in cancer, the expression/activity of several SG components are often deregulated in cancer cells, thus likely modulating their ability to adapt to stress conditions usually affecting transformed cells within tumors or metastatic cells. However, whether SG formation and functions display an oncogenic or tumor-suppressive role in cancer cells remains debated. In this regard, the ability of SGs to counteract cellular senescence and apoptosis by sequestering PAI-1 [17] and pro-apoptotic factors, such as TRAF2, or RACK1, may contribute to cancer cell survival [18]. Intense efforts aiming at characterizing the mRNA/protein content of these granules further uncovered numerous cancer-related factors suggesting that SGs may have the ability to sequester and/or stabilize the mRNA of oncogenes and tumor suppressors, thus affecting their impact on carcinogenesis [12]. Finally, whether SG formation can render cancer cells more resistant to various anti-cancerous approaches (e.g., chemotherapy, radiotherapy) remains to be clearly demonstrated but is supported by the ability of various anti-cancer molecules, e.g., sorafenib, one of the few drugs available for the treatment of advanced HCC, to trigger SG assembly [19]. Based on these observations, the concept emerges that modulating the formation of SGs with specific molecules may thus represent a potential therapeutic approach alone, or in combination with other treatments (i.e., chemotherapy, radiotherapy, etc.). In this review, we discuss our current knowledge on the function of SGs and their main components in HCC development, as well as currently available molecular tools targeting SGs for therapeutic purposes.

## 2. Molecular Bases and Complexity of SG Biogenesis

SGs are membrane-less cytoplasmic compartments made of ribonucleoproteins complexes and exhibiting a liquid-like property, which allowing the rapid exchange of mRNAs and proteins with the cytosol. The mechanisms regulating the dynamic assembly/disassembly of SGs are complex but appear to be widely conserved across species [20]. SG formation is triggered by a variety of cellular stresses including nutrients deprivation, hypoxia, heat shock, UV irradiation, oxidative and ER stresses, impaired protein degradation, and many others [21]. Specific circulating mediators (e.g., prostaglandins PGJ2/PGA1, oxidized-low density lipoproteins LDLs) or dietary factors such as obesogenic diets were also shown to promote SG formation in mouse macrophages and liver tissues [22], thus suggesting an important impact of dietary habits and chronic inflammatory/metabolic diseases on SG formation. As well, several anti-cancerous treatments, e.g., sorafenib, oxaliplatin, carbonyl cyanide p-(trifluoromethoxy) phenylhydrazone, or radiotherapy, trigger SG assembly in various cancer types (see Mahboubi et al. [23] for the list of anti-cancer molecules triggering SG assembly), which appears to protect them from death, leading to the concept that SG formation may represent an important survival mechanism for cancer cells [12].

**Figure 1:**
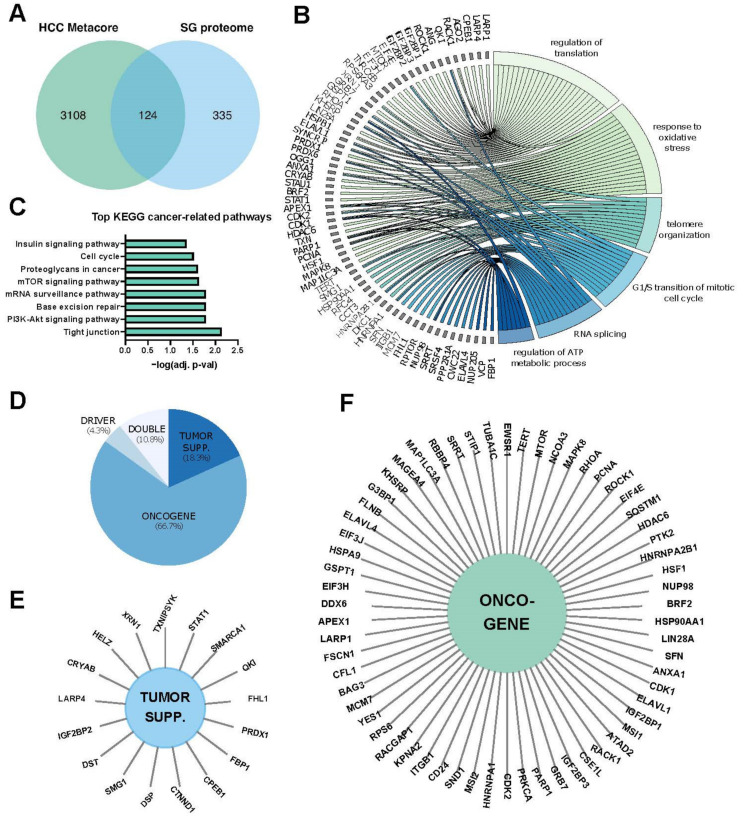
The SG proteome. (**A**) The stress granule (SG) proteome (https://msgp.pt/, accessed on 1 April 2021) was crossed with genes associated with hepatocellular carcinoma (HCC) obtained with the Metacore database (https://portal.genego.com/, accessed on 1 April 2021). (**B**) Gene ontology (GO) enrichment for biological processes (BP), with a cutoff of adjusted *p*-value at 0.05 was performed on the 124 genes found in (**A**). A chord-plot highlighting the most relevant processes and genes was constructed using the GOplot R package [24]. (**C**) KEGG pathway enrichment of 124 genes at a cut-off of 0.05 of the adj. *p*-value. (**D**) Annotation of cancer functions of the 124 genes using CancerMine [25]. (**E**,**F**), Sun diagrams showing annotated tumor suppressors (**E**) and oncogenes (**F**).

More than 400 proteins have been identified in isolated SGs by proteomic-based approaches (http://rnagranuledb.lunenfeld.ca/, accessed on 1 April 2021). About 50% of them are RNA-binding proteins (RBPs), while the others are involved in a wide range of cellular processes (e.g., metabolism, stress responses and cancer-related processes) (Figure 1). The composition of the SG proteome may, however, probably depend on several factors including the genetic context (i.e., mutations) or environmental factors (e.g., dietary factors, physical activity, inflammation). Structurally, SG are not uniform and are composed of internal dense structures referred to as “cores” that can be biochemically purified and contain a high amount of RNA and proteins. These cores are surrounded by a less concentrated shell, termed the “dynamic shell”, allowing a dynamic exchange of mRNPs with the cytosol or other cytoplasmic compartments (e.g., P-bodies) [20].

**Figure 2:**
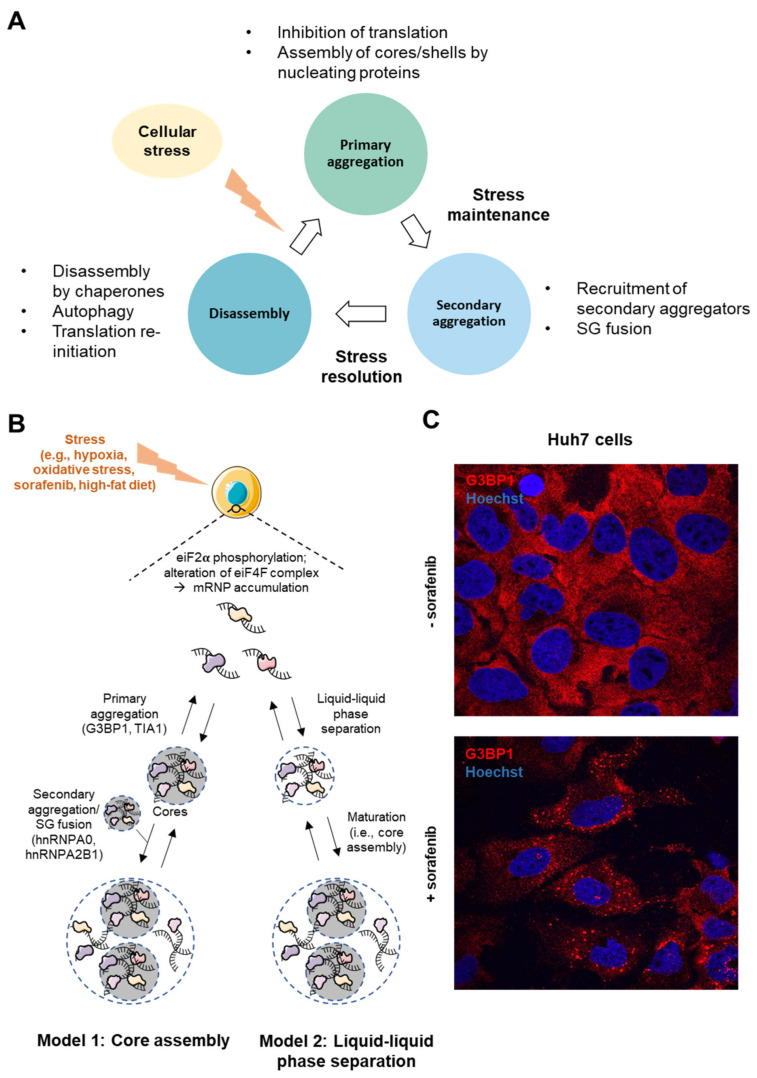
Stress Granule assembly. (**A**) The cycle of stress granule formation and disassembly. (**B**) SGs in the liver can form during stressful events such as hypoxia, oxidative stress, sorafenib treatment or high-fat diet. Two models of SG assembly have been described. In the “core first” model, nucleating proteins (e.g., G3BP1 and TIA1) form a stable core, and later, other SG-associated proteins are recruited to form the dynamic shell. Alternatively, in the ‘LLPS first’ model, proteins bound to transcripts assemble through interactions of their IDD domains. Further on, highly dense fractions form SG cores. (**C**) Confocal microscopy images of SG formation (G3BP1 staining in red; Hoechst-33342 staining in blue) in hepatic Huh7 cancer cells after 24 h treatment with 5 μM sorafenib (63× magnification).

The precise mechanisms and temporal/structural sequences of SGs biogenesis are still not fully understood but two models have been proposed to date. In both models, the first step appears to start with eIF2α phosphorylation by various kinases (e.g., PERK, eIF2α kinase 3, Protein Kinase R), which inhibits its activity, thus preventing the formation of eIF2/GTP/tRNAi ternary complex and triggering the dissociation of mRNAs from polysomes [12,21,26]. eIF2α-independent mechanisms initiating SG formation have also been proposed such as the alteration of the eIF4E or eIF4F complex, which promotes cap-dependent translation [26,27]. Then, two variations of the subsequent steps leading to SG assembly have been proposed (Figure 2):

1. In the “core first” model, the increased pool of untranslated mRNAs is bound and oligomerized by RBPs (e.g., G3BP1, TIA1, FMRP) bearing either a prion-like domain (PLD) or an intrinsically disordered domain (IDD), necessary for the recruitment of other proteins. This step, forming stable core structures is called “primary aggregation” [20]. The PLD and IDD domains of RBPs are enriched in glycine and uncharged polar residues (i.e., asparagine, glutamine, serine), which promote electrostatic interactions and liquid–liquid phase separation (LLPS)” [28]. Due to these special biophysical properties, SGs behave as hydrogel-like structures and are often considered as “viscous liquid droplets”. Then, the recruitment of additional ribonucleoproteins with weaker interactions (e.g., hnRNPA0, hnRNPA2B1, EWSR1) contributes to the formation of a dynamic shell (secondary aggregation) [20]. During the primary aggregation step, the transport of RBPs within SGs requires functional microtubules and motor proteins (i.e., dyneins and kinesins) [29,30]. Consistent with the role of microtubules in this process, HDAC6, which is a microtubule-associated deacetylase, reduces tubulin-α acetylation (Lys40) and promotes SG formation [31]. Finally, when the stress persists, other SGs components are recruited, allowing the growth and fusion of SGs in a process called “coalescence”, wherein several cores are embedded in a dynamic shell.
2. In a second model called “LLPS First”, oligomerization of mRNAs with proteins containing IDD is believed to promote LLPS. Then, in a further step, the high density of core components stabilizes the core structures, which are assembled inside LLPS.

Finally, reversibility of SG assembly is ensured by several clearance mechanisms proposed to be mediated by (i) autophagy, (ii) translation re-initiation, (iii) chaperone proteins, (iv) mRNA decay in processing bodies (P-Bodies), and/or (v) proteasome-dependent degradation of SG proteins.

The dynamic of SG assembly is tightly regulated by specific signaling pathways, such as the PI3K/AKT or the p38/MAPK pathways [32], which induce the assembly of SG components by activating the S6 Kinases-1 (S6K1), a downstream effector of mTORC1 signaling, which in turn phosphorylates and inhibits eiF2α [33]. mTORC1 was also shown to phosphorylate and activate 4E-BP1, which binds and inhibits the eIF4E initiation complex [34]. Of note, mutations and/or non-genomic alterations in cancer, often lead to the constitutive activation of these signaling pathways, which are strong promoters of SG formation. Various post-translational modifications (e.g., acetylation, phosphorylation, methylation) of key SG components were further reported to also govern the dynamic formation of functional SGs in pathological conditions [14,35,36,37].

Based on our current knowledge, it is likely that there is still much to be discovered about the pleiotropic mechanisms, functions, and relevance of SGs assembly in the cellular physiology and diseases. However, abnormal formation and functions of SGs occur in hepatic diseases and likely HCC, as supported by the altered expression of specific SG components, deregulated signaling pathways involved in SG assembly, as well as a deficient mechanism of SGs clearance, which are observed in these diseases.

## 3. SGs in Hepatic Carcinogenesis

**Figure 3:**
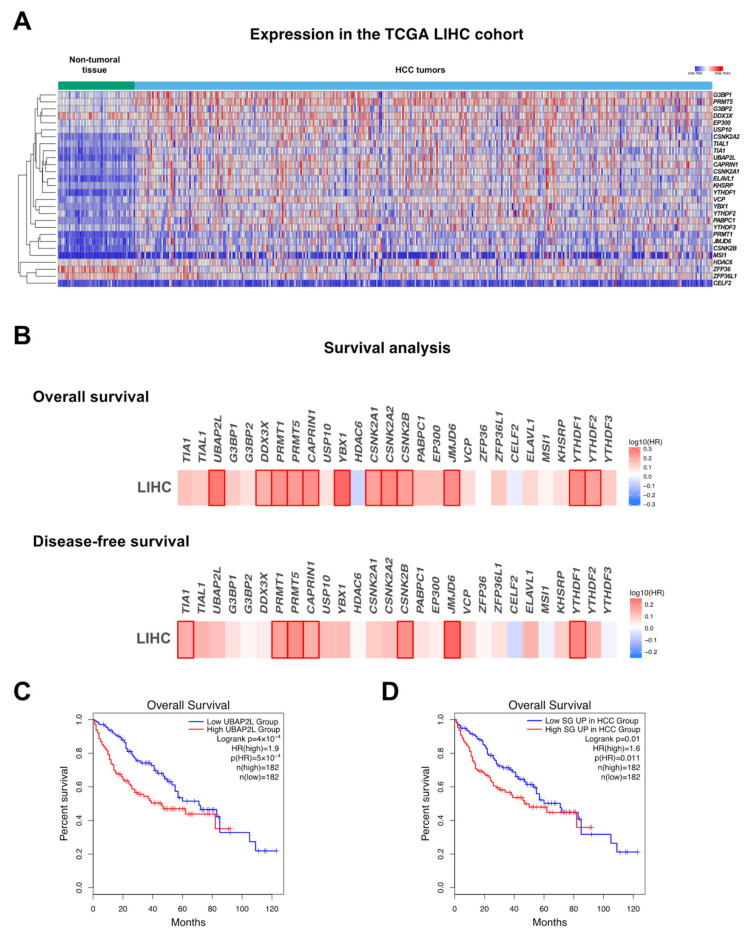
SG assembly in hepatocellular carcinoma. (**A**) mRNA expression levels of genes associated with stress granules in the LIHC TCGA cohort. (**B**) Heat map showing hazard ratio (HR) for overall and disease-free survival based on the expression of genes associated with stress granules in the LIHC TCGA cohort (http://gepia.cancer-pku.cn/, accessed on 1 April 2021). Positive hazard ratio indicates lower possibility of survival. Frames indicate significance. (**C**) Survival curve showing overall survival of patients expressing high vs. low levels of UBAP2L. (**D**) Survival curve showing overall survival of patients expressing high vs. low levels of SG-associated genes that are significantly upregulated in the LIHC TCGA cohort.

As previously discussed, SG formation is increased in many types of cancer cells, where they are believed to potentially exert an oncogenic function. However, an oncogenic role of SGs in cancer was not firmly demonstrated but is rather deduced from a set of observations, mostly derived from in vitro experimental approaches, such as bioimaging analyses, gain and loss of function analyses of specific SG components, or biochemical isolations and characterization of SG-containing subcellular fractions. As illustrated in Figure 1, approximately one third of transcripts/proteins present in SGs is functionally involved in classical cancer-related processes, thus supporting a significant role for SGs in carcinogenesis. In HCC particularly, only a few studies have provided correlative links between SGs biogenesis and hepatic carcinogenesis. This includes evidence showing that in vitro hepatic cancer cell resistance to sorafenib, a multi-kinases inhibitor used for the treatment of advanced HCC, is associated with the formation of SGs [19] and the observation that SG formation in HCV-infected hepatocytes is required for an efficient viral replication [38]. Furthermore, key factors intimately involved in the SG biogenesis or RBPs located in SGs and controlling the expression of hepatic metabolism, inflammation, and cancer-related genes [39] are significantly deregulated in hepatic diseases and cancer (Figure 1 and Figure 3A). As well, oncogenic signaling pathways typically overactivated in HCC (e.g., PI3K, p38/MAPK) were shown to drive SG formation in cells. Importantly, whether SG formation and presence is increased in vivo in HCC animal models or in patients with HCC was never firmly established in contrast to other cancer types. In the following sections, we discuss key factors and processes regulating SG biogenesis and function that are significantly altered in hepatic diseases and cancer and which may potentially affect the onset and/or the progression of these pathologies.

### 3.1. Nucleic Acids and Proteins Involved in SG Formation

**Figure 4:**
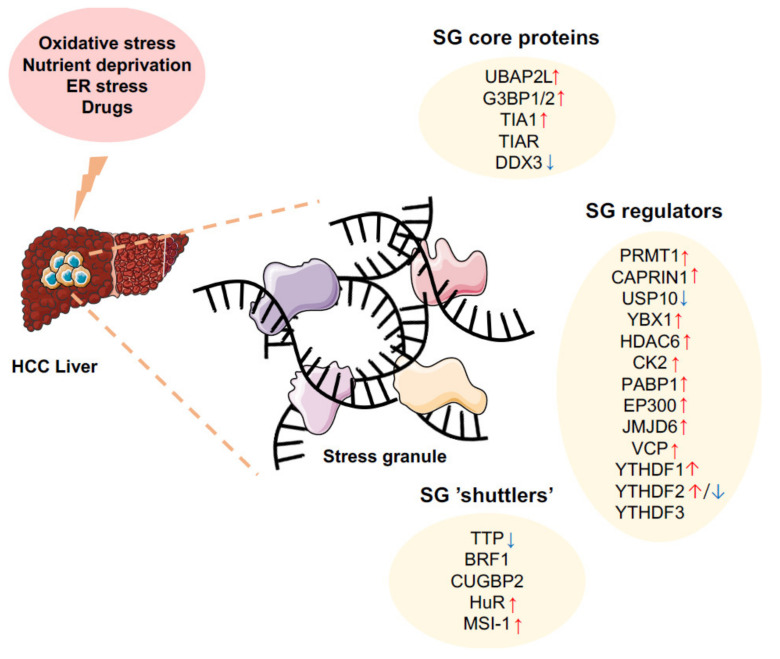
Alterations of SG components in hepatocellular carcinoma. Hepatocellular carcinoma is associated to the alteration of several SG components (core proteins, SG regulators, and SG shuttlers). Together, these alterations may importantly contribute to SG formation upon cellular stresses (e.g., oxidative stress, nutrients deprivation, ER stress, drugs). SG may in turn alter cell survival and death, angiogenesis, and other cancer-related processes. Arrows indicate higher (red) or lower (blue) expression in HCC based on literature (see respective paragraphs).

Overexpression of specific factors involved in the aggregation phase of SG formation is sufficient per se to trigger SG assembly, even in the absence of any cellular stress, while reducing their expression or activity considerably prevents SG formation in stress conditions [12,13]. Deregulated expressions/activities of these SG nucleators by various mechanisms are frequently observed in HCC (Figure 3A and Figure 4). Below are discussed key factors implicated in SGs biogenesis and for which experimental evidence suggests that they might be associated with the onset and development of HCC.

#### 3.1.1. UBAP2L (Ubiquitin-Associated Protein 2-Like)

UBAP2L is involved in the ubiquitin-proteasome pathway, but functions also as an essential component of SG assembly. It contains several specific domains including a ubiquitin-associated domain (UBA) required for the binding to ubiquitin chains [40,41] and an Arg-Gly-Gly (RGG) motif allowing the recruitment of other SG components. Another domain of UBAP2L, called the domain of unknown function (DUF), has poorly known functions but appears to be necessary to form complexes with other SG nucleators, such as G3BP1/2 (discussed in the next section) [40]. UBAP2L can, however, form SGs independently of G3BP1/2 [42] and its expression is sufficient per se to trigger SG formation, even in absence of any cellular stress [43,44]. UBAP2L has oncogenic properties in various cancers [43,44], but in the liver, its role is poorly defined and UBAP2L was never associated to SG formation. Nevertheless, emerging evidence indicates that the overexpression of UBAP2L in HCC correlates with a poor clinical outcome [45,46]. In vitro studies further indicate a tumor promoting function of UBAP2L through its ability to promote SMMC-7721 hepatocarcinoma cell proliferation, survival, and migration/invasion [45,47]. The RGG-containing domain can undergo various post-translational modifications, which regulate SG assembly. For example, PRMT1, a protein arginine methyltransferase overexpressed in HCC, promotes carcinogenesis, but in the meantime, methylates UBAP2L on the RGG motif, thereby inhibiting SG formation [40,48,49,50] and thus questioning the necessity to assemble SGs for the oncogenic functions of UBAP2L.

#### 3.1.2. G3BPs (Ras GTPase-Activating Protein-Binding Proteins)

The G3BP family has three members (i.e., G3BP1, G3BP2a, and G3BP2b), which interact with the SH3 domain of the Ras GTPase activating protein (RasGAP) and promote Ras signaling [51]. As for UBAP2L, G3BPs lack a PLD, but appear to act as key regulators of SG assembly [52]. The RNA recognition motif (RRM) of G3BPs allows their interaction with the 40S ribosomal subunit, while an RGG domain mediates mRNA binding [51]. G3BPs promote SG assembly through poorly characterized mechanisms triggering interactions with other SG components such as USP10 or CAPRIN1, which inhibits and promotes SG formation, respectively [53]. Of note, CAPRIN1 is overexpressed in HCC and correlates with a poor prognosis [54,55,56], while USP10 is downregulated in HCC and possesses various tumor suppressive functions [57]. Most of available studies are focused on G3BP1, which is frequently upregulated in a variety of cancers, where it seems to exert an oncogenic function [58]. Recent findings showed that RBPs, such as the Y-box binding protein (YBX1), which is overexpressed in many cancers including HCC [59,60], can upregulate G3BP1 expression by promoting its translation [61]. In HCC, G3BP1 induction was shown to contribute to cancer cells migration by increasing SLUG expression [62], but whether this was associated with increased SG formation was not investigated.

G3BPs are tightly regulated by post-translational modifications. Among them, phosphorylation of G3BP1 on Ser^149^ by Casein Kinase-2 (CK2) inhibits SG formation, as evidenced in osteosarcoma cells (U20S) [63]. CK2 is frequently overexpressed in HCC, correlates with a poor clinical outcome [64] and triggers various carcinogenic processes, including cell proliferation [65], resistance to death stimuli [66], and cancer cell migration/invasion [67]. Moreover, CK2 is also involved in hepatitis delta virus (HDV) replication [68] and NAFLD by promoting SIRT1 phosphorylation [69]. Acetylation of G3BP1 represents another regulatory mechanism of SG assembly. This regulation is mostly mediated by the CBP/P300 acetylase and the histone deacetylase 6 (HDAC6), which, respectively, inhibits and promotes SG assembly through acetylation/deacetylation of G3BP1 on lysine 376 (K^376^). Acetylation of K^376^ impairs G3BP1 interaction with its partners (e.g., USP10, CAPRIN1 or PABP1) and thus SG assembly [35]. Surprisingly, CPB/P300, HDAC6 and PABP1 are all upregulated in human HCC and correlate with a poor prognosis (Figure 2A,B) [70,71,72]. Finally, other post-translational modifications of G3BP1 can also modulate SG assembly, such as methylation by the oncogenes PRMT1 and 5, which are highly expressed in HCC and impair SG assembly [48,49,50] or demethylation by JMJD6 (Jumonji domain-containing 6), a tumor-promoting histone arginine demethylase favoring SG assembly and also overexpressed in HCC [37]. How these highly complex and antagonistic post-translational modifications of G3BP1, which are strongly deregulated in HCC, impact SG formation and functions in cancer cells was never experimentally investigated and outcomes in terms of formation of functional SGs in HCC cells remain purely speculative. Finally, the role of other G3BPs (2a and 2b), which may compensate or synergize with G3BP1, was likely underestimated in previous studies, and deserves further consideration [73].

#### 3.1.3. T-Cell-Restricted Intracellular Antigen-1 (TIA1)

TIA1 is an important SG component, which binds to AU-rich sequences in the 3′UTRs of its target transcripts through three RRMs (RNA recognition motifs) [74]. TIA1 contains a PLD in its C-term required for its self-aggregation but also likely for interactions with other SG components. During cellular stress, TIA1, together with other co-factors (e.g., TIA1-related protein, TIAR), sequesters target mRNAs into SGs, where they are kept translationally silent [74]. TIA1 localization, and therefore presence in SGs, can be regulated through the control of its nuclear-cytoplasmic shuttling, where nuclear accumulation is Ran-GTP-dependent, while its export is Chromosomal Maintenance 1 (CRM1)-dependent [75]. However, whether this mechanism is important for SG formation in liver cells is currently unknown.

TIA1 is mostly considered as a tumor suppressor, due to its ability to reduce the translation of transcripts promoting carcinogenesis (e.g., cyclooxygenase-2, COX-2) in many cancers. Accordingly, TIA1 expression is frequently downregulated in human cancers and its loss correlates with a poor prognosis [76,77]. However, in the liver, TIA1 could exert a dual function since it appears also to behave as an oncogene. TIA1 mRNA expression is indeed upregulated in HCC and hepatic cancer cells [78] and can act as an oncogene due to its ability to silence the tumor suppressor IGFBP3 [79,80]. Such oncogenic activity remains to be confirmed in vivo as well as whether TIA1 is required for SG formation in hepatic cancer cells. Nevertheless, the activity of TIAR, which is an important co-factor of TIA1 involved in SG assembly, is inhibited in HCC by *PHAROH* lncRNA, thereby promoting MYC translation [81]. Whether this oncogenic effect is related to an impaired SG assembly remains, however, to be investigated.

#### 3.1.4. DDX3 (DEAD-Box RNA Helicase 3 or CAP-Rf)

DDX3 is a ubiquitously expressed protein, which possesses ATPase and helicase activities and is involved in mRNA splicing and transcription [82]. Its helicase core contains two Recombinase A (RecA)-like domains, both of which display specific motifs responsible for RNA binding (reviewed in [83]). In Hela cells, DDX3 was shown to inhibit translation through its binding to eIF4E and PABP1 (Polyadenylate-Binding Protein 1) and thus to favor the first step of SGs formation [82]. This function was independent of its ATPase/helicase activity. Consistent with the role of SGs in this process, silencing of DDX3 importantly reduces SG formation and renders HeLa cells more sensitive to death stimuli [82]. In the liver, DDX3 appears to exert a tumor suppressive function in the liver [84] by promoting p21 upregulation in hepatic cancer cells or by repressing stemness [85]. Upon HCV infection, DDX3 interacts with the 3′UTR of HCV RNA and IKK-α and redistributes them into SGs [38], which then colocalize with the HCV core around lipid droplets. DDX3 functions importantly contribute to the HCV life cycle since silencing of DDX3 was shown to impair HCV replication. Moreover, DDX3 plays an important role in the HCV life cycle by interacting with the viral non-structural proteins NS5A and YBX1, as evidenced in Huh7.5.1 cells [86]. However, whether this function requires the formation of SGs is currently unknown. Surprisingly, whereas DDX3 is strongly downregulated in HBV-induced HCC, this is not the case in HCV-positive patients [87].

#### 3.1.5. G4DNA (G-Quadruplex DNA Structures)

G4DNA are quartets of guanine organized as a planar ring and linked by hydrogen bonds [88]. Recent findings indicate that G4DNA structures promote SG formation in the context of oxidative stress and DNA damage, as evidenced in melanoma cells treated by hydrogen peroxide. Once formed, these structures are exported to the cytosol and interact with RBPs (e.g., TIA1, TIAR, YBX1, HuR). Similarly, to protein nucleators, overexpression of G4DNA is sufficient to trigger the formation of SGs [88]. It thus appears that G4DNA are important cellular factors involved in SG formation and cancer-related cellular processes, but their role in HCC has currently been not investigated.

#### 3.1.6. tRNA-Derived Stress-Induced RNAs (tiRNAs)

tiRNAs are a class of non-coding RNAs, which displace the eIF4F complex from the m^7^GTP cap, thereby impairing cap-dependent translation and SG assembly [89]. This function has been observed with 5′-tiRNA but not 3′-tiRNA and relies on a 5′ terminal oligoguanine motif (5′-TOG), which fold in G-quadruplex structures [90]. This can interact with the translational silencer YBX1 via its cold shock domain [90]. tiRNAs are generated by angiogenin in harmful conditions by the cleavage of mature tRNAs within the anticodon loop [89]. Increasing evidence indicates that angiogenin is overexpressed in HCC and promotes cancer cells proliferation, migration, tumor vascularity, and EMT [91,92]. Whether these effects are linked to SG remains to be demonstrated, but it is likely that angiogenin overexpression may contribute to G4DNA synthesis and thus to SG formation.

#### 3.1.7. m^6^A RNA-Related Proteins

N^6^-methyladenosine (m^6^A) is the most common RNA modification [93]. It regulates mRNA stability, splicing, and translation [94] and can promote the recruitment of modified RNAs to stress granules in response to oxidative stress [94]. RNA methylation is coordinated by a “writer” complex, composed of the methyltransferase-like 3 (METTL3), methyltransferase-like 14 (METTL14), and the Wilms’ tumor 1-associating protein (WTAP), while demethylation is enabled by an “eraser” complex composed of fat mass and obesity-associated protein (FTO) and AlkB homologue 5 (ALKBH5) [94]. A third class of proteins called “readers” recognize methylated RNA and can impact their fate. YTHDF1–3 proteins, for instance, bind to m^6^A RNA and interact with SG components (i.e., G3BPs) [95]. Recent studies show that YTHDF proteins contribute to stress granule formation and that SGs are enriched in m^6^A RNAs [96], but whether “readers” or other m^6^A RNA-related proteins regulate the formation of functional SGs was not investigated in HCC. However, alterations of their expression are frequently observed and correlate with a poor clinical outcome [97]. In particular, several m^6^A RNA-related proteins with relevant function in cancer were found upregulated in HCC, thus potentially contributing to SG assembly. These include (i) METTL3, which increases the glycolytic capacity of HCC cells [98]; (ii) WTAP, which promotes hepatic cancer cells proliferation and tumor growth in vivo [99]; and (iii) the demethylase FTO, which fosters HCC development [100]. Other members of these families were found on the contrary to be downregulated in HCC, such as METTL14 [101,102] and ALKBH5 [97,103], and to exert a tumor suppressive function. Alterations of “readers” were also uncovered in HCC, such as the upregulation of YTHDF1, which increases cell proliferation and migration [104,105,106,107]. YTHDF2′s role in liver cancer is still controversial, with some data showing its downregulation and consequential decreased cell proliferation during hypoxia in HCC cells lines [108], while others reported an upregulation of YTHDF2 in HCC correlating with a poor survival [109]. Finally, YTHDF3 has not yet been investigated in the context of liver cancer; however, in silico analyses do not demonstrate any unequivocal deregulation in HCC tumors (Figure 3). However, whether YTHDF members affects SG formation and function in liver cancer still remains obscure.

### 3.2. Mechanisms Regulating SG Clearance in HCC

Defective clearance of SGs may importantly contribute to their accumulation in cancer cells. Considering these potential mechanisms of SG clearance, the accumulation of SGs in cancer cells is again paradoxical. Indeed, autophagic fluxes are usually increased in HCC and contribute to cancer cell survival and resistance to therapeutic molecules (e.g., sorafenib) [19]. Moreover, the components of the HspB8-HSP70-Bag3 complex, which contribute to SG disassembly by promoting autophagic-dependent degradation of misfolded proteins, are frequently overexpressed in HCC [110]. The Valosin-containing protein (VCP/p97), an ATPase belonging to the AAA family (ATPase-associated with diverse cellular activities), also triggers SG disassembly by interacting with ubiquitinated proteins and promoting their degradation [111] but also by fostering autophagosome maturation [112]. Accordingly, alterations of VCP expression in several diseases (e.g., Paget disease, inclusion body myopathy) lead to an impairment of autophagy and SG accumulation. However, in HCC, VCP is upregulated, and its silencing reduces hepatic tumor progression in vivo [113]. Finally, kinases activating VCP and thus reducing SGs, such as ULK1 and ULK2 (Unc-51-like Kinase1/2) [114], are also upregulated in HCC tumors [115].

Based on these observations, it is currently unclear how SGs are stabilized in an environment favoring their clearance, indicating that additional but still unknown mechanisms regulating SG biogenesis and degradation remain to be uncovered.

### 3.3. RNA-Binding Proteins Controlling mRNA Stability/Translation

RBPs are major components of SGs and important regulators of gene expression. Among them, AU-rich element binding proteins (AUBPs) govern the mRNA stability and translation of 5 to 8% of the transcriptomes by binding to AU-rich elements present in the 3′UTR of target mRNAs [116,117,118,119]. AUBPs usually regulate the fate of target mRNAs by recruiting them within either P-bodies or SGs. Importantly, the expression/activity of several AUBPs is altered in pre-cancerous hepatic stages and HCC, thereby modulating the expression of cancer-related transcripts including those of key factors governing metabolic and inflammatory processes (Figure 2A and Figure 3). Below, the most important AUBPs and other RBPs present in SGs are discussed and are reported to play a significant role in HCC development.

#### 3.3.1. TTP

TTP is encoded by *ZFP36.* It contains a zinc finger domain with a double zinc finger motif (Cys-Cys-Cys-His) responsible for RNA binding, three quadruple proline motifs responsible for the binding of the 4EHP (a.k.a. eIF4E2) -GYF2 (GRB10-interacting GYF protein 2) cap-binding complex, and a Not-1 binding domain [120,121,122]. It is usually described as a tumor suppressor, downregulated in many human cancers, and its level correlates with a poor clinical outcome [123,124,125]. TTP binds to mRNA transcripts and targets them for degradation in P-bodies. In physiological conditions, TTP is associated to P-bodies, whereas, upon stress, it can colocalize within SGs, as evidenced in Hela and COS7 cells [126,127]. It is therefore described as a protein shuttling between these two entities. TTP was proposed to be recruited to SGs upon specific cellular stress such as FCCP (carbonyl cyanide p-trifluoro-methoxyphenyl hydrazone)-induced energy deprivation (mitochondrial oxidative phosphorylation uncoupling) [126]. Translocation to SGs was prevented by MK2 kinase, which promotes TTP phosphorylation and sequestration by 14-3-3 protein, as evidenced in COS7 cells [126]. TTP was also reported to promote P-body and SG fusion [127]. In the liver, we recently showed that TTP promotes the development of non-alcoholic steatohepatitis (NASH), a condition from which HCC can develop [5]. In human HCC, TTP is downregulated at the protein level, and we could show using transgenic mouse models that TTP displays a dual oncogenic and tumor-suppressive role depending on the stage of the disease [125,128]. TTP overexpression was further shown by others to prevent activation of LX2 stellate cells suggesting that TTP may also restrain hepatic fibrosis development depending on the cell context and environment [129]. Different mechanisms regulating the expression/activity of TTP might in part be responsible for the different and sometimes antagonistic roles of TTP in liver pathologies. Indeed, suppression of TTP phosphorylation by MK2 inhibitor was shown to impair TTP activity in HCC cell lines [130], whereas TTP ability to regulate c-Myc was abrogated by methylation of a single CpG site in its promoter [131]. We also recently showed that the TTP expression is regulated by the HNF4α/EGR1 axis in Huh7 cells [125]. Although the precise role and functions of TTP in liver diseases and HCC have started to be delineated, whether TTP regulates SGs biogenesis or requires these entities for its functions, as suggested in non-hepatic cell lines [127], remains to be investigated in the liver.

#### 3.3.2. BRF1 (Butyrate Response Factor 1, *ZFP36L1*)

BRF1 is encoded by *ZFP36L1* and is a member of the ZFP36 family. Similarly to TTP, BRF1 binds to mRNAs through a tandem zinc finger domain with a double zinc finger motif [132,133], it promotes the mRNA decay of various cancer-related transcripts (e.g., VEGFA), and its expression is reduced in several cancers [132]. BRF1 was reported to decrease cell proliferation in a cyclin D1-dependent manner in colorectal cancer cells [134], and to regulate the expression of various mRNAs involved in hypoxia and cell cycle regulation (e.g., HIF1A, E2F1, etc.) in bladder cancer cells [135]. BRF-1 may also importantly participate to SG formation, as its overexpression can also trigger their assembly [127] but only fragmentary information is available about its role and functions in the liver. In hepatic cancer cells and primary hepatocytes, overexpression of BRF-1 was reported upon ethanol exposure, thus suggesting that it may contribute to ALD development and potentially HCC [136]. BRF1 was also suggested to regulate the bile acid metabolism, since it promotes the decay of CYP7A1 mRNA [137]. Similarly, to TTP, evidence indicates that BRF1 activity is regulated by phosphorylation. Protein kinase B (PKB) was shown to phosphorylate BRF1 on Ser^203^ and Ser^92^ phosphorylation in HIRc-B and mouse embryonic fibroblasts, thus inducing its binding to 14-3-3 and disruption of BRF1-dependent mRNA decay [138]. As for TTP, whether BRF1 and SG biogenesis/functions are linked in hepatic cells is currently unknown.

#### 3.3.3. HuR

HuR is encoded by *ELAVL1* and is an RNA-binding protein belonging to the embryonic-lethal abnormal vision in the drosophila (ELAV) family [139]. HuR is ubiquitously expressed and usually localized in the cell nucleus from where it translocates to the cytosol to control translation/stability of target mRNAs. The protein contains two tandem RRMs and a hinge region followed by a third RRM [140]. The hinge region is subjected to various post-translational modifications, including phosphorylation by various kinases within the HuR nucleocytoplasmic shuttling domain, which regulates the localization of the protein [140]. The stabilizing property of HuR on various mRNA transcripts relies on its ability to compete with or displace destabilizing factors, including miRNAs but also other AUBPs (i.e., TTP) sharing the same ARE binding site. HuR is overexpressed in several cancers including HCC and correlates with a poor clinical outcome [139,141,142]. The overexpression of HuR in cancer cells importantly promotes carcinogenesis by fostering the overexpression of oncogenes and the loss of tumor suppressors [141,142]. In stress conditions, HuR accumulates in SGs and promotes the stabilization of various oncogenic transcripts, thereby favoring cancer cell survival [143]. In the liver, HuR is upregulated in HCC patients and increases the expression of transcripts involved in cell cycle regulation (i.e., cyclin A and D1) [144], inhibits apoptosis through direct interaction with the *FAS* mRNA and by inhibiting caspase-3 activity [144,145], and facilitates hepatocyte de-differentiation by stabilizing the *MAT2A* transcript [146]. HuR is also an important promoter of sorafenib-induced ferroptosis, a type of cell death that can lead to fibrosis development in the liver [147]. However, despite the importance of SG formation in sorafenib resistance [19], no specific association between HuR and SG formation was reported in the liver. The regulation of HuR expression/activity has already been extensively reviewed elsewhere [140] and points to a strict control of its cellular localization and transcript binding, two processes that are also intimately linked to stress granule formation.

#### 3.3.4. CUGBP2

CUGBP2 is encoded by *CELF2.* This RNA-binding protein contains three RNA recognition motif domains and modulates alternative splicing and mRNA translation. For instance, it regulates COX-2 mRNA translocation to stress granules in myoblastic H9c2 rat cells [148]. CUGBP2 expression is reduced in many cancers; however, its role in human HCC remains to be examined. Nevertheless, CUGBP2 was shown to regulate, together with APOBEC, C to U editing of apolipoprotein B (APOB), which is a protein important for sterol metabolism, i.e., VLDL, LDL, and HDL formation in the liver [149]. Of note, accumulation of cholesterol in hepatocytes fosters NAFLD progression toward NASH and HCC [150]. Inactivation of APOB is observed in human HCC and correlates with a poor prognosis [151]. However, whether the editing activity of CUGBP2/APOBEC significantly impact cholesterol metabolism in HCC cells remains to be demonstrated. The mechanisms of CUGBP2 regulation are poorly know, but its alternative splicing (i.e., exon 14 inclusion, which encodes for the first half of the third RRM) promotes the alternative splicing of insulin receptor transcript in HeLa cells [152]. The existence of such mechanisms is, however, currently unknown in the liver.

#### 3.3.5. Musashi-1 (Msi-1)

Msi-1 is another important RBP promoting mRNA translation inhibition and decay. It contains two RRMs and a PABP binding domain [153]. Msi-1 expression is upregulated in a variety of cancers and promotes tumor development, due to its ability to control the expression of specific cancer-related factors (e.g., oncotachykinin) [154]. In the liver, little information is available, but recent studies indicate that Msi-1 is upregulated in HCC tissues as compared to non-tumoral adjacent tissues [155]. Furthermore, the overexpression of Msi-1 in hepatic cancer cells enhances cell proliferation [155]. This effect was associated with the ability of Msi-1 to downregulate the tumor suppressor APC, thereby triggering activation of the β-catenin pathway [155]. In stress conditions, Msi-1 is localized in SGs and contributes to chemoresistance, as evidenced in colorectal cancer [156], but such a functional link remains to be demonstrated in HCC.

## 4. Are SG Potential Therapeutic Targets in HCC?

Although it is far from clear whether SGs contribute to cancer survival and chemoresistance in HCC, studies in other cancers argue that this might indeed be the case [12,157]. Targeting the assembly of functional SGs may therefore represent a novel therapeutic approach to at least restore chemosensitivity of HCC towards drugs currently approved such as sorafenib.

### 4.1. Targeting SG Nucleators

Targeting SG nucleators may represent an efficient strategy to prevent SG assembly and thus potentially re-sensitize hepatic cancer cells to physiological death stimuli and anti-tumoral therapies (e.g., sorafenib) [19]. Moreover, given the reported roles of SGs in hepatic virus replication [158] targeting their assembly represents an alternative strategy to treat HCV and HBV infections [86,158]. The therapeutic virtues of various compounds affecting the expression/activity of SGs nucleators have been examined for various diseases (Table 1). In this regard, pharmacological inhibitors of SG nucleators are a promising strategy, although the effects of only a few of these compounds have been investigated in hepatic diseases. Alternatively, the delivery of siRNAs (i.e., aptamers) to reduce the expression of these SGs nucleating proteins, i.e., G3BP1, may represent an additional approach as demonstrated in colorectal cancer (CRC) [159]. Resveratrol [160], or epigallocatechin-gallate (EGCG) [161] were, for example, reported to reduce G3BP1 expression in lung (H1299 and CL13) cancer cells [162,163], while attenuating NAFLD [164] and restraining HCC development [165]. However, given the pleiotropic effects of these compounds in the cells, how G3BP1 inhibition contributes to their anti-tumoral activity remains to be evaluated. Small peptides inhibiting G3BP1 activity, such as GAP161, can also block SG assembly, as evidenced in colon cancer [58], but still not in HCC. Similarly, other compounds such as EMICORON or RHPS4, two G-quadruplex ligands or angiogenin inhibitors (e.g., chANG) can efficiently prevent SG formation [166,167], but their effects in HCC remain to be demonstrated. More recently, restoring DDX3 expression with rottlerin, a natural compound derived from *Mallotus Philippinensis* [168], or diosgenin [169] have been indicated for HCC treatment. However, if SGs are confirmed to mediate chemoresistance and tumor recurrence, the use of these compounds in clinic should be considered with care since DDX3 clearly promotes SG formation.

### 4.2. Targeting Regulatory Pathways Involved in SG Assembly

Several pathways have been involved in the regulation of SG assembly. Among them, the AMPKα/mTORC1 pathway may represent an appealing target, as SG formation is usually associated to a decrease of mTORC1 and an activation of AMPKα [32]. Therefore, molecules activating or inhibiting mTORC1 or AMPKα may abrogate or promote SG assembly. In agreement, Compound-C, an inhibitor of AMPKα, prevents SGs induced by cold exposure in yeast [21] and displays anti-cancerous properties [181] (Table 1).

However, the role of mTORC1/AMPKα signaling on SG assembly is still unclear and controversial, as other studies have documented that mTORC1 activation is necessary for SG assembly in breast cancer [182]. In addition, in the liver, inhibition of the mTOR pathway with rapamycin-derived compounds to fight HCC is poorly effective [183]. Finally, the effect of AMPKα activators (e.g., metformin, or physical activity, which possess important anti-tumor properties in HCC [184,185]) on SG assembly, remains to be investigated.

### 4.3. Targeting Post-Translational Modification of SG Components

As discussed before, both HDAC6 and SIRT6 promote SG assembly [186]. HDAC6 inhibitors may therefore represent a potential strategy to impair SG formation in HCC. Among the HDAC6 inhibitors (e.g., A452 [171], C1A [187], ACY-1215 [173], MPT0G612 [174], several possess strong anti-tumor properties [188], but whether this effect is linked to an impairment of SG assembly is currently unknown (Table 1). Similarly, for SIRT6, very few inhibitors have been developed (e.g., OSS_128167) [175,189] and although they display anticancer properties (e.g., large B-cell lymphoma) [175], their impact on hepatic cancer cells is currently unknown.

### 4.4. Increasing SG Clearance

Favoring clearance of SGs may represent another relevant approach to limit SG accumulation in cancer cells. As already mentioned, the clearance of SGs is partially mediated by autophagy [190]; increasing autophagic flux in cancer cells may therefore lower the amount of SGs and re-sensitize cancer cells to chemotherapy. In the liver, autophagy regulates multiple functions, including lipid metabolism, insulin sensitivity, hepatocytes cell death, or sorafenib resistance in HCC [191,192]. In HCC, autophagy is currently considered as a survival mechanism of cancer cells [193]; therefore, several autophagic inhibitors have been developed and proposed for HCC treatment in preclinical models [194]. However, the impact of autophagy inhibition on SG dynamics is currently unknown and should be carefully considered given the potential role of SGs in HCC cells survival and tumor recurrence.

### 4.5. Targeting Microtubules

Functional microtubules and motor proteins are required for the transport of RNPs during SG assembly [29]. Destabilizing microtubules by pharmacologic approaches (e.g., vinblastine, nocodazole) efficiently prevents SG assembly [29,30]. On the contrary, stabilizing molecules, such as paclitaxel, trigger SG formation. In HCC, the combination of vinblastine with temsirolimus (an mTOR inhibitor) displays anticancer properties [176]. However, the impact of such a combination on SG dynamics remains to be investigated, as well as the real importance of SG modulation by these compounds as compared to other strongly affected cellular processes [195].

### 4.6. Targeting AUBPs Associated with SGs

Several AUBPs control the stability and translation of transcripts involved in inflammatory, metabolic, or cancer-related processes. Targeting these proteins may therefore represents a potential therapeutic option. Although these proteins were originally considered “undruggable”, specific inhibitors of HuR (e.g., DHTS, MS444) have been developed and inhibit HuR activity by interacting with its RNA-binding domains in the case of DHTS and by inhibiting its relocation to the cytoplasm, thereby enabling transcript association with P-bodies, in the case of MS-444 [178,180]. Interestingly, DHTS suppresses the growth of HCC cell lines through the JAK2/STAT3 pathway [179]. MS-444 has not yet been tested in liver diseases nor HCC. Given the anticancer properties of these molecules [196], investigating the precise mechanisms involved in their anti-tumoral activity and how the latter is related to SGs biogenesis deserves in-depth investigations prior to consideration for clinical applications.

Except for a few compounds targeting key components of SGs specifically (e.g., G3BP1, HuR, see Table 1), a number of drugs and clinically approved therapeutics have been found to affect, although mostly indirectly, the expression/activity of SG components or key regulators of these latter, which have been described in previous sections of this review. Most of the drugs/therapeutics described in Table 1 have well recognized anti-tumoral properties and have been documented to affect a wide variety of cellular cancer-related processes. However, to which extent inhibition of SG formation, or functional impairment of specific SG components, contribute to the anti-tumoral effects of these drugs/therapeutics remains unknown to date. Further investigations of the role and functions of SGs in HCC are therefore required prior to envisage any therapeutic interventions targeting these structures specifically. Finally, once the presence or absence of SGs in cancer cells, including HCC, has been firmly validated, their relevance as prognostic/diagnostic biomarkers for cancer risk, staging, and chemoresistance should also be evaluated and thus may represent potential biomarkers of chemoresistance, as suggested for sorafenib in HCC [19].

## 5. Conclusions

Although the formation of SGs has been associated with cell survival and resistance to anti-tumoral treatments in several cancers, the role of these cytoplasmic entities in HCC remains enigmatic. Several key components of the SGs, implicated in their nucleation and functions, are strongly deregulated in hepatic cancer cells, suggesting that both the formation and functions of these entities are modulated with cell transformation and the tumor microenvironment. However, based on our still fragmentary knowledge about the roles and functions of SG components and their involvement in mechanisms governing the formation and integrity of SGs, whether they represent a structural hallmark of cancer cells, e.g., in the liver, remains an open question. Several factors considered as key elements of SGs still have a poorly characterized function in HCC and most of them likely also exert part of their functions independently of their presence in mature SGs. In addition, it is probable that several factors exert redundant functions in nucleating and maturing SGs, which renders the analyses of the role of these structures in pathophysiological situations more complex. In HCC, the expression of SG components is clearly altered, and their functions appears to be regulated by multiple and complex antagonistic mechanisms, as well as the formation of mature SGs. In addition, most of the studies performed to date were using in vitro hepatic cell systems, which could be irrelevant as compared to the in vivo pathophysiological situation both in animal models of HCC or in patients. Future analyses should therefore first clearly investigate whether SG formation occurs in precancerous stages of the liver and/or preferentially in transformed hepatocytes as compared to normal cells. Then, additional work will be required to precisely understand how SG components regulate their assembly in a single entity and whether the liquid-phase organization of these SG components in the cytoplasm is required for their functions. Finally, the current dogmatic view of SGs as oncogenic cytoplasmic entities promoting chemoresistance and cell survival must be taken with caution, at least in the case of liver cancer. Verification of this hypothesis should set the basis for future efforts aiming at targeting SGs for therapeutic purposes or using these structures as biomarkers to predict cancer development stages and therapeutic outcomes.

## Figures and Tables

**Table 1:** Drugs/therapeutics targeting SG components and key regulators of SG assembly.

Molecule	Target	Cell Models	Tested in HCC Cells *
Resveratrol	G3BP1 [163]	SK-MEL-5 human melanoma, HCT116 human colorectal carcinoma	yes [170]
EGCG (epigallocatechin-gallate)	G3BP1 [162]	H1299 and CL13 lung cancer cells	yes [165]
GAP161	G3BP1/2 [58]	HCT116 human colorectal carcinoma	no
EMICORON	G4DNA [167]	BJ EHLT immortalized human fibroblasts, A90-LUC colorectal murine cells	no
chANG	Angiogenin [166]	HT1080 (human fibrosarcoma), HM7 (human colorectal carcinoma), NIH/3T3 (Mouse fibroblast)	no
Rottlerin	DDX3 [168]	QGY7703, SMMC7721 liver cancer cells	yes [168]
Diosgenin	DDX3 [169]	HepG2, SMMC-7721 human liver cancer cells	yes [169]
Compound-C	AMPKα [21]	COS7 cells monkey kidney fibroblasts	no
A452	HDAC6 [171]	Multiple myeloma cells: MM.1S, H929, BM-MSCs, PCS-500-012 cell lines	no
C1A	HDAC6 [172]	Panel of cancer cell lines (colon, breast, endometrial, epidermal, lung, myeloma, neuroblastoma, ovarian, and prostate cancer cells)	no
ACY-1215	HDAC6 [173]	Lymphoma cells: OCI-LY10	no
MPT0G612	HDAC6 [174]	Colon cancer cells: HCT-116, HT-29 and DLD-1	no
OSS_128167	SIRT6 [175]	Large B-Cell Lymphoma: DLBCL cells	no
Vinblastine	Microtubules [30]	CV-1 green monkey kidney fibroblasts	yes [176]
Nocodazole	Microtubules [30]	CV-1 green monkey kidney fibroblasts	no
Paclitaxel	Microtubules [30]	CV-1 green monkey kidney fibroblasts	yes [177]
Temsirolimus	mTOR inhibitor [176]	Hep3B, HepG2, Huh7	ys [176]
DHTS	HuR [178]	Colon cancer cells, HCT116	yes [179]
MS-444	HuR [180]	Colon cancer cells, HCT116	no

* This column indicates if the listed molecules have already been tested on HCC cells and does not necessarily indicate if the molecules are able to affect the targets in HCC.

## Data Availability

The Stress granule proteome (Figure 1) was obtained from https://msgp.pt/, accessed on 1 April 2021. Genes associated to hepatocellular carcinoma (HCC, Figure 1) were obtained with the MetaCore database (https://portal.genego.com/, accessed on 1 April 2021). Expression of SG components and survival information in HCC patients (Figure 3) were obtained from the CancerLivER database (https://webs.iiitd.edu.in/raghava/cancerliver/index.html, accessed on 1 April 2021) and the Gepia database (http://gepia.cancer-pku.cn/, accessed on 1 April 2021), respectively.

## Abbreviations

ALD: alcoholic liver disease; AMPK: AMP-activated protein kinase; ARE: AU-rich Element; ATX-2: ataxin 2; AUBP: Adenylate-Uridylate-rich elements binding protein; Bag3: BCL2-associated athanogene 3; BRF1: Butyrate response factor 1; CK2: casein kinase 2; COX: cyclooxygenase; CUGBP2: CUG triplet repeat-binding protein 2; DDX3: DEAD-Box RNA helicase 3; DUF: domain of unknown function; DYRK3: Dual Specificity Tyrosine Phosphorylation Regulated Kinase 3; ELAV: embryonic-lethal abnormal vision in drosophila; ER: endoplasmic reticulum; G3BP: Ras GTPase-activating protein-binding protein; GO: gene ontology; HBV: hepatitis B virus; HCC: hepatocellular carcinoma; HCV: hepatitis C virus; HDAC: histone deacetylase; HDV: hepatitis delta virus; HSP70: heat shock proteins 70; HspB8: Heat Shock Protein Family B (Small) Member 8; IDD: intrinsically disordered domain; JMJD6: Jumonji domain-containing 6; KEGG: Kyoto encyclopedia of genes and genomes; KSRP: K-homology splicing regulator protein; LDL: low density lipoprotein; LLPS: liquid–liquid phase separation; miRs: microRNAs; Msi-1: Musashi; mTORC1: mammalian target of rapamycin complex 1; NAFLD: non-alcoholic fatty liver disease; ONC: oncogenes; PABP1: Polyadenylate-Binding Protein 1; P-Bodies: Processing-Bodies; PI3K: phosphoinositide 3-kinase; PLD: prion-like domain; PRMT: protein arginine methyltransferase; PTEN: Phosphatase and TENsin homolog; RACK1: Receptor for Activated C Kinase; RBP: RNA-binding protein; RGG: Arginine-Glycine-Glycine; RNP: ribonucleoprotein; RRM: RNA-recognition motif; SG: stress granule; SIRT6: sirtuin 6; TDP-43: TAR DNA-binding protein 43; TIA1: T-cell-restricted intracellular antigen-1; TIAR: TIA-1-related; TTP: tristetraprolin; UBA: ubiquitin-associated domain; UBAP2L: Ubiquitin-associated protein 2-like; ULK: Unc-51-like kinase; USP: Ubiquitin Specific Peptidase; UTR: Untranslated Region; VCP: Valosin-containing protein; VEGF: Vascular endothelial growth factor; YBX1: Y-box binding protein
